# Childhood Prevalence of Involvement with the Child Protection System in Quebec: A Longitudinal Study

**DOI:** 10.3390/ijerph20010622

**Published:** 2022-12-29

**Authors:** Tonino Esposito, Johanna Caldwell, Martin Chabot, Anne Blumenthal, Nico Trocmé, Barbara Fallon, Sonia Hélie, Tracie O. Afifi

**Affiliations:** 1 École de Travail Social, Université de Montréal, Montréal, QC H3T 1J4, Canada; 2 School of Social Work, University of Michigan, Ann Arbor, MI 48109, USA; 3 School of Social Work, McGill University, Montreal, QC H3A 0G4, Canada; 4 Factor-Inwentash Faculty of Social Work, University of Toronto, Toronto, ON M5S 1A1, Canada; 5 Institut Universitaire Jeunes en Difficulté, Montreal, QC H2L 4R5, Canada; 6 Department of Community Health Sciences, University of Manitoba, Winnipeg, MB R3T 2N2, Canada

**Keywords:** child protection, childhood lifetime prevalence, survival table analysis, child neglect

## Abstract

The goal of this study, the first of its kind in Canada, was to estimate the child lifetime prevalence of child protection involvement in Quebec. Using administrative and population data spanning 17 years, we performed a survival analysis of initial incidents of child protection reports, confirmed reports, confirmation of a child’s security or development being compromised, and placement outside the home for one day or more. We found that before reaching the age of 18 years, over 18% of children were reported to child protection at least once, one in every ten children (10.1%) in the province had a report that led to the finding of their security or development being compromised, and over 5% were placed outside the home. We found that neglect was a primary concern in close to half (47.6%) of cases. By using a full population dataset, we obtained a more accurate prevalence estimate than studies using synthetic cohort life tables. These findings only captured initial incidents of involvement with child protection, meaning this study does not show the extent of recurrent involvement for some children. The findings reflect prior results showing that neglect is common in initial child protection involvement but less pervasive than has been shown in incidence studies, suggesting that recurrent child protection involvement is more driven by neglect than initial incidents are.

## 1. Introduction

### 1.1. Importance of Understanding Child Protection Involvement

North American child protection systems intervene exceptionally in families when there are concerns regarding the family’s ability to maintain a minimum level of caregiving. When children and their families become involved with child protection systems, it can be a sign of significant challenges within the household or a lack of supportive conditions in the broader environment around them, and, more often, a complex combination of these factors [1,2]. Annual counts of child protection cases in North America suggest that a majority of cases relate to neglect [3,4,5]. Past studies also driven by cases labeled as neglect have suggested that families are often chronically involved in child protection [6]. Illustrating the scope of involvement with child protection systems across childhood, particularly when this involvement may be chronic and recurring, is crucial for identifying ways to reduce reliance on exceptional child protection intervention.

The significant clinical and social impacts of childhood maltreatment require better understanding—both in terms of scope and severity—to inform relevant preventative policies and community-based interventions [7]. While child protection involvement is legally confined to children and their families, the negative impacts of child maltreatment are often not confined to childhood [8]. Indeed, research shows maltreatment can have increased negative impacts on mental and physical health both in youth and into adulthood [9,10,11,12,13,14,15,16,17,18,19,20,21]. The implications of child protection involvement for society more broadly are also significant: studies examining the cost of child abuse and neglect suggest an order of billions of dollars per year may be spent when considering social services, health, education, employment, judicial, and personal costs [22,23,24,25]. However, a child protection intervention paradigm that prioritizes intervening for children’s safety rather than intervening to support children and families can ignore opportunities for preventative services [26]. One way to identify opportunities for reducing child protection intervention is to describe, at a population level, the cumulative risk of child protection involvement across the developmental stages of childhood.

The current study aims to provide a fuller picture of the cumulative risk of experiencing child protection involvement by computing the first Canadian prevalence estimates of child protection involvement in Quebec—the first study of its kind in Canada. Using administrative data from Quebec spanning the years 2000 to 2017 and a life table analysis approach, we determined an estimate of the childhood prevalence (age 0–17 years) of reported maltreatment, confirmed reports, substantiated reports, and out-of-home placement. With these analyses, we aim to (a) provide a clearer picture of the situation in Quebec such that preventative policymaking and practice may consider the cumulative, long-term risk of child protection involvement [26], (b) provide a template for other Canadian jurisdictions that could support cross-jurisdictional analysis, and (c) encourage concerted efforts at the pan-Canadian level to use child protection administrative data to support longitudinal analysis of the scope of child protection across Canada. By beginning to estimate the prevalence of involvement with child protection in this province, we can obtain a fuller view of the extent of this experience and illustrate questions for further research.

### 1.2. Prior Estimates of the Scope of Child Protection Involvement

Typically, studies attempting to understand the scope of child protection involvement in families look at annual rates within a child population or use a point-in-time survey or administrative data. Recent annual reports from the United States in 2020 suggest that among the child population that year, 2.89% of children had a screened-in report, 0.84% had a substantiated report, and 0.22% of children entered foster care [27]. While there are no analogous pan-Canadian annual reports, some provinces and territories publish their yearly data. In Quebec, the focus of the present study, the Director of Youth Protection (DYP) does report annually on various figures related to child protection cases. These annual provincial reports suggest around 2.5% of all children each year have a child protection report that is retained for further investigation, 0.96% have a report leading to a finding that the child’s security or development is compromised, and 0.76% of children are placed outside their homes for child protection reasons [28,29,30,31,32].

Periodic surveys in Canada provide another set of findings regarding the incidence of involvement with child protection systems. The most recent Canadian Incidence Study of Reported Child Abuse and Neglect (CIS-2019) estimated 4.8% of children were investigated for child protection reports, 1.64% of children had reports substantiated, and 0.24% of children were placed outside the home during the investigation. Incidence studies have also been conducted periodically in certain provinces in Canada [33]. In Quebec, the most recent study of the incidence of child protection involvement (First Nations/Quebec Incidence Study of Child Maltreatment and Serious Behaviour Problems Investigated by Child Protection Services in 2019; FN/QIS-2019) found that 2.36% of children investigated for child protection reports, and 0.24% of children were placed outside the home during investigation [34]. Recently, the Ontario Incidence Study of Reported Child Abuse and Neglect (OIS, 2018) found that 6.29% of children had a child protection investigation, 1.61% had a substantiated report of maltreatment, and 0.02% of children were placed out of the home during the investigation stage [35]. These findings are only comparable for illustrative purposes, as each jurisdiction has its own child protection legislation, definitions, regulations, and data collection processes. Within jurisdictions, the above annual and survey rates are useful for providing a descriptive, retrospective, year-to-year snapshot of child welfare activities within a population over a certain time period, which can be useful for monitoring child protection systems from a management and budget allocation perspective.

However, incidence estimates are unable to capture the percentage of children who experience maltreatment over the course of their childhood and accordingly cannot support analysis of long-term outcomes nor can they inform effective prevention and support for well-being. Measuring childhood prevalence rather than simply measuring incidence allows for a more complete and longer-term illustration of the range of experiences of child protection involvement in certain geographies. Rather than simply providing annual maltreatment rates across large jurisdictions, research on prevalence provides actionable information for targeted policymaking [36]. While prevalence studies are widely used in other fields, such as public health, it is only recently that prevalence studies have been applied to child protection research [5]. With improvements in the technological capacity to capture and analyze large data sets, many child maltreatment prevalence studies drawing on administrative data in the United States have emerged in the past five years [5,37,38,39]. To date, there have been no prevalence studies of child protection involvement published in Canada.

Differing methodological approaches to capture the prevalence of childhood maltreatment have led to variations in the way the problem can be understood across jurisdictions [40,41]. The unit of analysis is one methodological choice that is often constrained by available data: When prevalence studies include all siblings or caregivers in a household, prevalence estimates will be higher than if only child-level data is included (e.g., [39]). Maltreatment experienced over the course of childhood has typically been studied using retrospective studies of adults [42,43]. For example, a recent study in Canada used self-report surveys of adults (*n* = 23,395) to estimate the childhood prevalence of child abuse, finding the prevalence of any type of child abuse (i.e., not including neglect) to be 32% among respondents [9]. While self-report measures with adults are one way to retrospectively estimate childhood prevalence of maltreatment, they are subject to recall bias: Individuals who struggle as adults are more likely to report having experienced maltreatment. Longitudinal studies using child protection administrative data are more reliable but less common due to the increased rigor of data collection and study length required. Importantly though, as Wildeman and colleagues note, the prevalence rates determined by administrative data rely only on cases that were reported to child protection agencies, making the estimates of maltreatment low as the data inherently do not include unreported cases [5].

### 1.3. Previous Empirical Results of Prevalence Studies

Prior prevalence measures of childhood involvement with child protection systems show a range of findings across progressive involvement in these systems. In the United States, Kim and Drake estimated cumulative probabilities of maltreatment reports from age zero to 11 in 28 U.S. states and the District of Columbia, suggesting the likelihood for one report by age 12 was 32.41% [37], aligning with incidence studies suggesting child protection involvement is already experienced at high rates before adolescence [20] (Estimates of cumulative experiences of neglect in childhood also range widely depending on methodology and jurisdiction, but multiple studies suggest it is commonly experienced in earlier childhood. Kim and Drake (2019) [37] note in their recent study that neglect was the most common maltreatment type estimated for children aged zero to 11 for both initial and subsequent reports). Kim and colleagues used the U.S. National Child Abuse and Neglect Data System (NCANDS) file along with census data to estimate the prevalence of child welfare involvement by the age of 18, finding that 37.4% of all children experience a child protection investigation [38]. In the United States, Wildeman and colleagues conducted a prevalence study of substantiated maltreatment among children between 2004 and 2011 using NCANDS data (N = 5.5 million). Compared to the annual incidence of child maltreatment (estimated at about 1% of children in the U.S.), they found that 12.5% of all children in the study experienced substantiated maltreatment by the age of 18 years [44]. In a study on the lifetime risk of foster care placement, Wildeman and Emanuel found that up to 5.91% of all U.S. children were placed in foster care between ages zero and 18 [44].

Prevalence studies show a wide variation in the estimates of child protection involvement broken down by maltreatment type. As with incidence studies and annual reports, cross-jurisdictional comparisons of prevalence findings are limited by a lack of uniform definitions of maltreatment types and variation in legislation and practice norms. Though policies, legislative definitions, practice norms, and socioeconomic and cultural contexts shaping child rearing vary greatly across jurisdictions, some studies have examined the scope of different maltreatment types across multiple countries. In recent studies, estimates suggest that, globally, physical abuse may be experienced over the course of childhood by 22% to 28% of children [18,45]. Sexual abuse is also experienced at high levels when measured in terms of childhood prevalence: Barth and colleagues conducted a systematic review of 55 studies on childhood sexual abuse in 24 countries, finding a range of 8% to 31% prevalence for girls and 3% to 17% for boys [46]. In an analysis conducted in the U.S., Finkelhor and colleagues found that by age 17, 26.6% of girls and 5.1% of boys had experienced sexual abuse or sexual assault [47]. Hussey and colleagues also found in retrospective child maltreatment data from a cohort study of young adults (*n* = 15,197) that having been left alone as a child (which the authors interpret as possible supervision neglect) was the most prevalent at 41.5% of respondents, and 11.8% also experienced physical neglect [18]. Finally, in a review of a series of meta-analyses on global child maltreatment prevalence, Stoltenborgh and colleagues note that the overall estimated prevalence of emotional neglect was 18.4% and 16.3% for physical neglect [45]. These prevalence estimates, though variable, illustrate a larger proportion of the population that is impacted by child protection concerns across childhood (18 years) while incidence studies and annual findings examine a much shorter time period.

To the extent that budgeting for preventative child and family services in Canada can be based on evidence, what exists today are static estimates of maltreatment incidences that do not illustrate child protection involvement across childhood (e.g., CIS-1998; CIS-2003; CIS-2008; OIS-2018; FN/QIS-2019) [34,48,49]. Prevalence studies can begin to fill this gap to demonstrate the extent of involvement for children—specifically, the ages at which the risk of child protection concerns is particularly high and the types of child protection concerns that are more likely to emerge. In Quebec, a province-wide administrative child protection database exists that has allowed us to build a longitudinal dataset from 20 years of administrative case files. For this study, this rich dataset supported precise estimates of prevalence within the province that can be useful in child protection policy discussions.

### 1.4. Context of the Present Study

The policy context of child protection in Quebec has evolved over the past decades and is primed for more comprehensive data demonstrating the extent of child maltreatment. Major reforms in 2007 included changes intended to support permanency and stability for children involved with child protection, including implementing maximum time periods for out-of-home placement [50]. More recently, prompted by the death of a seven-year-old girl in 2019, a special commission was appointed to examine legal and practical aspects of the child protection system in Quebec. Following many months of expert testimony and contributions from the public, the commission report emphasized well-being rather than protection as the ultimate goal for children [51]. The findings of the report further highlighted the necessity of preventative services, particularly for children living in poverty, and that the child protection system ought to be a last resort rather than a mechanism for families to access needed services that could be obtained otherwise [52]. These findings align with other recent analyses in Canada proposing that child well-being be framed as the ultimate goal of child welfare and child protection systems [53] and reinforce questions about whether a safety focus is best when many child protection-involved children are not subject to protection concerns but rather chronic poverty and missing support. There is consensus in Quebec that change is needed to improve the child welfare system province-wide, but the evidence required to undergird informed changes has not been adequately demonstrated in research.

Accurately estimating the prevalence of child protection system involvement and the risk factors surrounding it can inform policy and practice decision making that is oriented to prevention and early intervention to minimize negative impacts on a substantial portion of the population. However, there are notable gaps in what the data has been able to demonstrate regarding the childhood prevalence of involvement with child protection systems in Quebec and across Canada more generally [4,26,54]. Despite the significant impact of child protection involvement on children, families, and communities, Canada lacks a robust body of child protection data nationally, particularly because there is not a national-level, representative data set in the country [55]. Given the existing data in many jurisdictions, child protection researchers are able to optimize data sets to answer complex questions through cross-sectional analysis but are unable to answer basic questions about child protection involvement among the general population. To date, there has not been a prospective child maltreatment prevalence study conducted in a Canadian jurisdiction or across Canada. The CIS, a cross-sectional survey conducted in 1998, 2003, 2008, and 2019 is currently the most comprehensive study of child protection surveillance data in Canada. While the CIS periodically captures annual estimates of reported, investigated, and substantiated maltreatment as well as investigation-stage out-of-home placement findings, in a representative sample of child welfare agencies as a survey measure, it does not provide a complete file of national administrative child protection data that would allow for the computation of prevalence rates. Unfortunately, while the CIS and some provincial incidence studies (e.g., OIS and QIS mentioned above) provide valuable insight into child protection trends, these incidence rates do not illustrate the scope of child protection involvement across the course of childhood.

Examination of involvement in child protection systems through a prevalence estimate rather than annual incidence rates is necessary if the goal is to illustrate the risk of involvement with child protection across childhood. This study relies on population data to calculate cumulative prevalence over a multiple-year-long period which expands its relevance beyond a point-in-time prevalence estimate. Our analysis aimed to fill a gap in empirical findings by asking to what extent child protection risk is present in the general child population. More specifically, the involvement with child protection systems for neglect reasons, seen through a prevalence lens, shows the extent to which chronic needs closely associated with socioeconomic vulnerabilities relate to the overall child protection prevalence risk for children, which in turn is magnified for certain marginalized populations [54,56]. Accordingly, in the present study, prevalence estimates are presented for neglect and non-neglect reports. We hypothesized that neglect would be significant but that its proportion of all initial child protection cases may be less than in incidence findings given the chronic, recurring nature of neglect illustrated in prior research.

Our unique longitudinal population dataset links investigation data to placement data, allowing for estimates of maltreatment-specific placement prevalence. In this study, we use the unique opportunity provided by our dataset to provide estimates of the lifetime risk of out-of-home placement according to the reason for initial investigation. Though others have used similar longitudinal administrative data for prevalence estimates [57,58,59,60], in this study, we provide the first known estimates of the lifetime risk of out-of-home placement for neglect and non-neglect-related cases. Thus, although our estimates are limited to Quebec, our analysis is significantly stronger methodologically and substantively than those produced with data from NCANDS and AFCARS due to the nature of our data, thus offering empirical and methodological implications relevant well beyond Quebec.

## 2. Materials and Methods

This longitudinal population study provides cumulative rates of childhood prevalence of involvement in Quebec’s child protection system using data from 2000 to 2017. In this section, we provide justification for our analytical approach; legislated definitions specific to Quebec that shape children’s trajectories in this system; descriptions of the analyses we conducted using SPSS 25 to estimate the prevalence of involvement in child protection by type of maltreatment and to estimate cumulative risk by age (0–17 years); and the sources of data used to conduct these analyses. The Canada Research Chair in Social Services for Vulnerable Children (950-232659) and an Insight Grant from the Social Sciences and Humanities Research Council (435-2018-0715) funded this study.

Our prevalence calculations offer methodological advantages compared to prevalence estimates produced with synthetic cohort single decrement life tables [5,41,47,59]. Single decrement (otherwise known as ordinary) life tables assume that the single attrition event (in this case, initial reports to child protection) happens only once during the event risk period. However, child protection intervention is an event that can occur multiple times during the event risk period. Multiple decrement lifetables may be used to handle this type of event but are not the appropriate tool to answer the question of what the probability of ever experiencing child protection intervention is. The fact that single decrement life tables assume a single attrition factor from any given observation is problematic for estimating lifetime prevalence of child protection intervention because many child protection datasets (such as NCANDS or AFCARS) simply do not have reliable data to identify that single attrition factor: initial first-time reports to child protection [60]. Synthetic cohort single decrement life tables used with this kind of unreliable data may thus overestimate initial reports by mistakenly recording an entry into the life table as an initial event when, in fact, it was a secondary, tertiary, or subsequent event. This overestimation can result in biased cumulative rates (see discussions in [36,58,59]). Our analysis is based on data from an actual population cohort of real individuals who were observed for up to seventeen years of the risk period (ages 0–17 years, from 2000–2017). Thus, our estimates are less biased and easier to interpret when compared to child protection intervention prevalence estimates based on a synthetic cohort life table approach.

As in other North American jurisdictions, child protection intervention in Quebec is meant to be exceptional. Concerns reported to the child protection system are funneled through a series of decision points that determine the nature and scope of the concern and what services or interventions may be necessary to “protect children whose security or development is or may be in danger” [61]. The provincial Youth Protection Act (YPA) defines these concerns as follows: abandonment, psychological ill-treatment, sexual abuse, physical abuse, serious behavioral disturbance, neglect, serious risk of physical abuse, serious risk of sexual abuse, and serious risk of neglect [61]. The definition of “neglect” differs from the other forms of possible concerns in that it refers to caregivers “failing” to meet a child’s needs rather than committing an act of maltreatment [61]. In Quebec and many other jurisdictions, neglect is associated with chronic poverty and other forms of unmet needs in families and communities rather than immediate protection concerns [4,62,63,64]. Accordingly, in this study, we distinguish between neglect and non-neglect forms of child protection concerns for some stages of child protection involvement. The designation of “risk of neglect” is included in the neglect category, as is risk of physical or sexual abuse in those categories.

**Figure 1:**
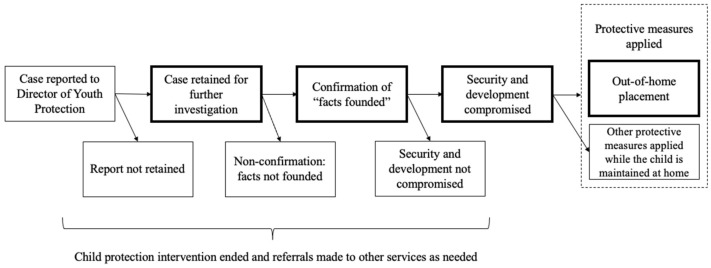
Overview of child protection decision points upon receipt of a report concerning a child in Quebec (bolded boxes indicate stages of focus in the present study).

When an initial report is received by the Director of Youth Protection (DYP), it is assessed according to whether at face value it fits the scope of concerns legislated by the Youth Protection Act. If it does, it is retained for further investigation. Then, the investigation examines the facts of the report to confirm the validity of the report, which leads to a confirmation of “facts founded” (FF). This step is unique in Quebec, as many other jurisdictions move to a substantiation decision directly after retention or “screening-in” of a report. Following confirmation of facts of the report, an evaluation of this confirmed report is evaluated to determine whether the child’s security or development is compromised (SDC), which is equivalent to a “substantiation” of the report as it is called in many other jurisdictions. In cases of substantiation, protective measures are implemented either voluntarily or ordered by the youth court. When concerns regarding the child’s security and development remain at risk with the child in the home, child protection authorities may consider out-of-home placement for a period of time [65]. Figure 1 highlights these stages of involvement in the child protection system after a report is received by the DYP (When this happens in Quebec, there is a range of potential alternative situations that may be implemented either voluntarily or under court order. If the report does not move forward in this trajectory (e.g., if the report is not retained, not confirmed, or child’s security/development is not compromised), the intervention will be ended, and referrals are made to other services if deemed necessary. It is important to note that while children may be removed for reasons other than child protection concerns, such as criminal justice involvement, our measurement of placement is limited to cases following a finding under the child protection system that a child’s security or development is compromised).

In this study, we estimate childhood prevalence of involvement with Quebec’s child protection system after an initial report for all children born between 2000 and 2017. First, through a survival analysis, we estimated cumulative risk of childhood prevalence in the following stages of involvement in child protection: (1) report retained for investigation, (2) confirmation of facts founded (FF), (3) security and development compromised (SDC), and (4) out-of-home placement following an initial finding of SDC. All estimates were adjusted down by 8% based on prior knowledge of duplicate files. Because the administrative data only captures a unique child identifier at the regional level rather than province-wide, duplicates arise due to mobility of the population between regions. Next, we broke down the estimates of child prevalence of confirmed and substantiated child protection reports according to whether the primary concern was specifically regarding neglect or another maltreatment concern. By examining neglect, which is not a commission of abuse or other active maltreatment and is representative of chronic need and poverty [4], there is a possibility of better understanding the extent of chronic family needs throughout childhood. Distinguishing between neglect and non-neglect maltreatment is important for understanding how and when families may end up under the purview of the child protection system after being failed by other needed, preventative services, which in turn can inform more targeted policy and resource allocation to fill these gaps. Finally, we estimated the probability of involvement in child protection at each age (0–17 years) based on the survival table built according to each age and risk of involvement at each stage (retained report, confirmed report, substantiated report, and placement). By estimating cumulative risk according to age, we were able to analyze the likelihood of involvement with the child protection system throughout childhood.

We obtained data for these analyses from multiple sources. Annual child population data for Quebec between 2000 and 2017 was drawn from holdings at the Ministry of Health and Social Services of Quebec (Ministère de la santé et des services sociaux; MSSS). These population data are based on actual Canadian census data (The Canadian census is conducted every five years) held at Statistics Canada which informs MSSS projections in between census years. Clinical administrative child protection data regarding child protection involvement over the life of the study was drawn from MSSS data holdings through a data sharing agreement. These data were denominated and include the following administrative data relevant for our analyses: child unique identifier, child age, and trajectories within child protection described above (reports to the DYP, confirmation of facts founded (for neglect and non-neglect), substantiation of reports (SDC; for neglect and non-neglect), and placement of the child outside the home). It is important to note that data on children’s ethnicities are not reliable in the child protection data across the province and subsequently was not included in the present analysis. Administrative child protection data were entered into the information system by frontline child protection workers.

## 3. Results

Survival table estimates of childhood prevalence of involvement with the child protection system in Quebec show that over 18% of children between ages 0 and 17 years are subject to an initially reported child protection concern that is retained for investigation. About 16% of all children had their reports confirmed, meaning the facts of these initial reports were founded (FF) prior to a determination of substantiation. Together, this means that more than half of retained reports (representing 10.1% of the child population) were substantiated. Of the substantiated reports for which the security or development was deemed compromised (SDC), more than one-third (representing almost 3.4% of all children) resulted in an initial placement outside the home. 

**Figure 2:**
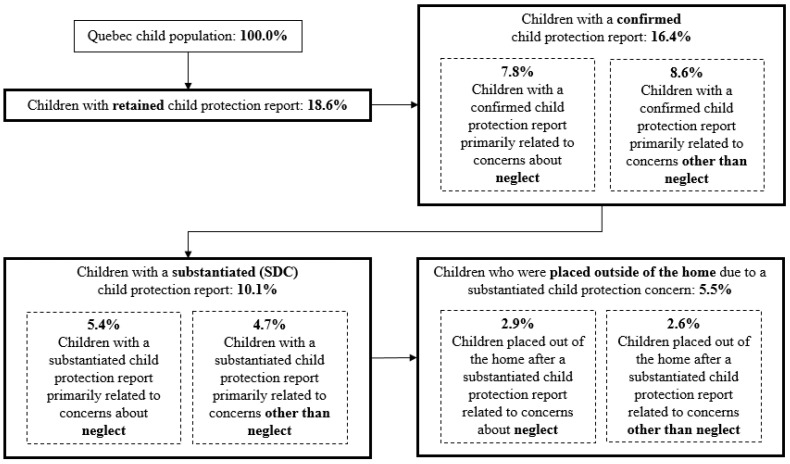
Survival table estimates of childhood prevalence of involvement in Quebec child protection system (as percentage of entire child population).

Further, we broke down the confirmed reports and substantiated reports according to whether the concerns about the child’s well-being were related to neglect or other non-neglect concerns about the child. For confirmation of the facts of a report, there were more reports related to non-neglect concerns, such as physical abuse, sexual abuse, or child behavioral challenges. However, slightly over half (5.4%) of reports that resulted in a finding of compromised security or development were related to neglect rather than other concerns. Children who were placed outside their homes after a finding of SDC had similar levels of neglect and non-neglect concerns. Based on these findings, the cumulative childhood prevalence of involvement with child protection in Quebec is illustrated in Figure 2 below. The percentages shown are a proportion of all children in the child population of Quebec.

The survival table analysis using data from 2000–2017 allowed for disaggregation of the findings above such that we could estimate the age at which children were first involved with child protection at the four stages we examined. To illustrate how these estimates relate to the cumulative risk of initial involvement with the child protection system over the course of childhood, we examined cumulative percentages over time using the age-disaggregated estimates for children’s first-time events between 2000–2017. Cumulative risk according to age is presented in Table 1.

Calculating the risk of involvement in the child protection system by age allowed for a full view of this phenomenon across childhood. Infants experienced the highest risk of retained, confirmed, and substantiated reports. The second highest risk at these stages was for fourteen- and fifteen-year-olds. Infants were the only age group to have over 1% of their population experience a substantiated report. The highest risk of placement outside the home was for teenagers between 13 and 16 years old at an average cumulative increase of 0.62% of each age category followed by infants at 0.44%. The findings for these age groups are explored in further detail in the Discussion section. 

## 4. Discussion

The findings of this prevalence study in Quebec illustrate the extent to which children in the province may become subject to exceptional intervention by the province due to concerns about their security or development. 

Prevalence studies have a unique role to play in understanding the scope of child protection involvement. While annual incidence studies are useful in providing a snapshot of new cases which can be compared to other years, this prevalence study used administrative data to view child protection involvement through a longer arc of time over the course of childhood. Compared to documented incidence rates of substantiated reports and out-of-home placement in Quebec, the estimated prevalence estimates of substantiation and placement found from our analysis were much higher. This is due to important methodological differences. For example, in 2019 the FN/QIS found that 1.69% of children had a substantiated report during that year [66] whereas we found that over the course of childhood, between 9.57% and 10.12% were found to have a substantiated report. Further, we estimate that about one-third (34%) of children with substantiated cases experience an out-of-home placement. By contrast, the proportion of placements among substantiated cases at the investigation stage in the QIS is about 37%. Compared to annual reports in the province during the same period of time [28,29,30,31,32], our prevalence estimates illustrate that a much higher proportion of the child population will be subject to a child protection report and substantiation and that they will experience a similar likelihood of placement following a substantiated report. These prevalence findings may be more compelling to policymakers wanting to understand the extent of placement risk children face and demonstrate the need to consider this method for the estimate depending on the policy objective.

Our estimates that about half of substantiated reports relate to concerns about neglect align with prior findings that neglect comprises a large proportion of child protection cases [3,4,5]. Importantly, this represents initial involvement with the child protection system and does not capture any subsequent child protection reports. This contrasts with prior findings which suggest that over 80% of child protection involvement relates to neglect, which does reflect recurrent reports, demonstrating the chronicity of neglect. We found that a slight majority of SDC findings had neglect as a primary concern, and almost half of cases that resulted in placement followed a neglect-driven SDC finding. These prevalence findings show neglect as less pervasive than has been shown in incidence studies, suggesting that recurrent child protection involvement is more driven by neglect and potential surveillance bias following initial reports. When neglect is often not a commission of maltreatment but rather an omission due to the chronic needs of these families [4,64], alternatives to child protection system intervention may be more preventative [7]. A few outlier jurisdictions with a low proportion of neglect cases have strong diversionary mechanisms away from child protection for children and families who may be best supported through other services [27]. Our findings, encompassing more data and a longer timespan than any prior study in Quebec or any Canadian province, reify the concern that a large number of children and families are subject to child protection intervention that may not address their underlying needs, compromising the extent to which child protection systems are truly equipped to improve child outcomes. This supports recent calls for more attention to prevention in child protection settings to better serve the needs of children [7]. When child protection involvement occurs without a clear protection concern present, many advocates suggest that community-based services and supportive programming will be more effective and desirable for vulnerable families [67,68,69,70].

Our findings regarding placement indicate that infants and adolescents are most likely to be removed from their homes. The high involvement of young children, especially infants, across the child protection stages we examined in this study may reflect the particular developmental risk of this age group [69]. For young children, emphasis on permanency and consistency for children in Quebec has brought attention to family preservation as a primary objective over time. More stringent timeframes ushered in by the reforms in 2007 may have pushed workers to avoid placement over time to avoid rigid timelines imposed by the permanency requirements [50].

Because Quebec has a decision point of confirming the facts of a report, this may provide for more decision points at the beginning of a case in which families may be diverted to more appropriate services (e.g., when there is a retained report and confirmation of facts is found but not a finding of compromised security or development). For example, situations of confirmation but not substantiation might include situations of interpersonal violence that ends because a perpetrator moves out of the house, a child who does not have an adequate lunch at school and appears dirty or unbathed is diverted from child protection to obtain other support, or a situation of physical abuse that is deemed not severe or repetitive and therefore not considered to be a threat to the child’s security or development. Because this level of decision making is not built into the majority of other jurisdictions’ processes when receiving reports, it is difficult to compare our findings to other jurisdictions, but the notable difference between the confirmed reports and the substantiated reports in the present study suggests that this level of granularity of decision making may be functioning to screen vulnerable families for other needs when child protection concerns are not high.

These findings demonstrate several possible ways that this and similar studies can inform preventative policy design that considers a longer view of the population’s vulnerabilities. For example, the extent of neglect-related cases undergird past calls for more supportive community-based family services, income supports, and differential responses to families reported to child protection [67,68,70,71,72]. The young age at which children are more likely to become reported to child protection systems also suggests that budget allocation to support families with infants could be more cost-effective. Together with other findings suggesting that recurrence is more likely for families initially reported to child protection systems for reasons of neglect [6], this study underlines the need for preventative support for families, especially while children are young.

These findings point to many more granular questions regarding the prevalence of child protection involvement that could inform policy improvements to support child well-being across the course of childhood. When the reasons for child protection involvement have been more expansively defined over time [73], the increasing awareness of vulnerability has not been matched by expanded preventative services. Given prior incidence studies in Quebec and other jurisdictions indicating variation across socioeconomic status in terms of who is most likely to become subject to a child protection report, prevalence studies may further illustrate the extent of this relationship. In addition, given prior findings regarding variation in child protection involvement across neighborhoods, understanding the prevalence (rather than incidence) of child protection involvement through this lens will help illustrate the scope of involvement such that appropriate supportive family services can be identified.

The implications of this study in terms of methodology are equally important as are the clinical findings and policy implications. The methodological differences between annual incidence and prevalence studies covering a period of time cannot be overstated. They are designed to perform very different functions: the former to provide a glimpse at new child protection involvement over one year and the latter to estimate the extent of this phenomenon over time—in this case, the course of childhood. It appears that when more data is available over a longer period of time, analyses may uncover higher levels of risk. This study undergirds the importance of ongoing efforts to improve the quality of the evidence base in child welfare settings such that more granular prevalence studies can be conducted—both across jurisdictions in Canada and at a national level. This prevalence study, the first of its kind in Canada, demonstrates the need for more data regarding who is likely to become involved with child protection.

Several limitations of this prevalence study must be noted. First, the available data limit the extent to which we can make claims based on our findings. As we are estimating counts for the latter years of the study period, we would expect to have more precise counts in a subsequent follow-up study with a longer time period including actual numbers. Further, because data regarding children’s ethnicity is inconsistently available in the Quebec administrative data, we could not include this variable in our analyses. This is a major limitation of the present study and many others, and we echo recent calls for better data regarding ethnicity in child protection [1]. While we focused on data regarding placement outside the home after an initial substantiated report, we did not include the length of time in out-of-home care—this means that this analysis does not distinguish between children placed for one night or six months. In addition, it does not capture placements that occurred following a second report to child protection. Finally, many children experience maltreatment without a report to child protection being made, meaning that data on child protection involvement, as is presented here, does not represent all experiences of maltreatment. These caveats suggest that our findings may underestimate the extent to which children experience placement outside the home.

Because child protection data in Quebec is captured at a child level, household experiences of child protection involvement (e.g., multiple children with child protection reports) cannot be gleaned from this study nor does this study capture recurrence of child protection reports. Basic estimates of child protection involvement do not capture many aspects of children’s individual situations: If there were multiple child protection concerns as is the case in many child protection reports, only the primary concern is captured in the data—this leaves the extent of different types of child protection concerns unknown. 

## 5. Conclusions

This study estimated the prevalence of children’s initial interaction with several stages of the Quebec child protection system across 17 years. Our findings suggest that a substantial proportion of the child population may become involved with the child protection system: Before reaching the age of majority, more than 18% of children will be subject to at least one report of child protection concern that is retained for further investigation. Further, 10% of the child population may have a child protection report that leads to a determination of compromised security or development, following which around more than 5% of all children may be placed outside the home for some period of time. Our estimates resulting from this study suggest that close to half of initial substantiated child protection concerns and subsequent placements relate primarily to neglect rather than other maltreatment concerns in Quebec. Compared to annual counts of child protection system involvement within this jurisdiction, these findings demonstrate a much more extensive risk that a given child will experience intervention by the child protection system at some point during childhood. These findings also suggest that a substantial amount of these reports may be diverted to more appropriate services. More analysis of the drivers of involvement in the child protection system can support improved prevention and provision of needed services.

## Figures and Tables

**Table 1:** Estimated cumulative risk of involvement in child protection in Quebec between 2000 and 2017 (age 0–17 years).

Age (Years)	Retained	Confirmed	Substantiated	Placed
0	1.78%	1.60%	1.14%	0.44%
1	2.78%	2.44%	1.65%	0.65%
2	3.77%	3.24%	2.13%	0.83%
3	4.78%	4.03%	2.61%	1.00%
4	5.77%	4.80%	3.05%	1.15%
5	6.82%	5.63%	3.51%	1.31%
6	7.91%	6.54%	4.02%	1.47%
7	8.99%	7.46%	4.55%	1.64%
8	10.03%	8.36%	5.06%	1.82%
9	11.03%	9.23%	5.55%	2.01%
10	11.96%	10.05%	6.02%	2.20%
11	12.88%	10.87%	6.49%	2.41%
12	13.80%	11.72%	7.01%	2.70%
13	14.87%	12.72%	7.68%	3.16%
14	16.15%	13.98%	8.55%	3.83%
15	17.32%	15.14%	9.35%	4.56%
16	18.21%	16.01%	9.92%	5.18%
17	18.59%	16.37%	10.12%	5.52%

## Data Availability

Metadata on the provincial child protection dataset used in this study can be found at https://www.msss.gouv.qc.ca/professionnels/documentation-sources-de-donnees-et-indicateurs/sources-de-donnees-et-metadonnees/pij/ (accessed on 12 January 2022).

## References

[1] Font S.A., Maguire-Jack J. (2020). The scope, nature, and causes of child abuse and neglect. Ann. Am. Acad. Political Soc. Sci..

[2] Garbarino J., Gelles R.J., Lancaster J.B. (2017). The consequences of child maltreatment: Biosocial and ecological issues. Child Abuse and Neglect: Biosocial Dimensions-Foundations of Human Behavior.

[3] Semanchin-Jones A., Logan-Greene P. (2016). Understanding and responding to chronic neglect: A mixed methods case record examination. Child. Youth Serv. Rev..

[4] Trocmé N., Kyte A., Sinha V., Fallon B. (2014). Urgent protection versus chronic need: Clarifying the dual mandate of child welfare services across Canada. Soc. Serv..

[5] Wildeman C., Emanuel N., Leventhal J.M., Putnam-Hornstein E., Waldfogel J., Lee H. (2014). The prevalence of confirmed maltreatment among US children, 2004 to 2011. JAMA Pediatr..

[6] Esposito T., Chabot M., Trocmé N., Fluke J.D., Delaye A., Caldwell J., Hélie S., King B., de la Sablonnière-Griffin M., Mackrell L. (2021). Recurrent involvement with the Quebec child protection system for reasons of neglect: A longitudinal clinical population study. Child Abus. Negl..

[7] Slack K.S., Berger L.M. (2020). Who is and is not served by child protective services systems? Implications for a prevention infrastructure to reduce child maltreatment. Ann. Am. Acad. Political Soc. Sci..

[8] Sugaya L., Hasin D.S., Olfson M., Lin K.-H., Grant B., Blanco C. (2012). Child physical abuse and adult mental health: A national study. J. Trauma. Stress.

[9] Afifi T.O., MacMillan H.L., Boyle M., Taillieu T., Cheung K., Sareen J. (2014). Child abuse and mental disorders in Canada. Can. Med. Assoc. J..

[10] Barker B., Kerr T., Alfred G.T., Fortin M., Nguyen P., Wood E., DeBeck K. (2014). High prevalence of exposure to the child welfare system among street-involved youth in a Canadian setting: Implications for policy and practice. BMC Public Health.

[11] Bell C.J., Foulds J.A., Horwood L.J., Mulder R.T., Boden J.M. (2019). Child abuse and psychotic experiences in adulthood: Findings from a 35-year longitudinal study. Br. J. Psychiatry.

[12] Bunting L., Davidson G., McCartan C., Hanratty J., Bywaters P., Mason W., Steils N. (2018). The association between child maltreatment and adult poverty–A systematic review of longitudinal research. Child Abus. Negl..

[13] Cyr K., Clément M.-È., Chamberland C. (2014). Lifetime prevalence of multiple victimizations and its impact on children’s mental health. J. Interpers. Violence.

[14] Fairbank J.A., Fairbank D.W. (2009). Epidemiology of child traumatic stress. Anxiety Disord..

[15] Felitti V.J., Anda R.F., Nordenberg D., Williamson D.F., Spitz A.M., Edwards V., Koss M.P., Marks J.S. (1998). Relationship of childhood abuse and household dysfunction to many of the leading causes of death in adults: The adverse childhood experiences (ACE) study. Am. J. Prev. Med..

[16] Font S., Dixon L., Perkins D.F., Hamilton-Giachritsis C., Craig L.A. (2017). Psychological, economic, and physical health consequences of child maltreatment. The Wiley Handbook of What Works in Child Maltreatment: An Evidence Based Approach to Assessment and Intervention in Child Protection.

[17] Hanson J.L., Knodt A.R., Brigidi B.D., Hariri A.R. (2015). Lower structural integrity of the uncinate fasciculus is associated with a history of child maltreatment and future psychological vulnerability to stress. Dev. Psychopathol..

[18] Hussey J.M., Chang J.J., Kotch J.B. (2006). Child maltreatment in the United States: Prevalence, risk factors, and adolescent health consequences. Pediatrics.

[19] Kisely S., Abajobir A.A., Mills R., Strathearn L., Clavarino A., Najman J.M. (2018). Child maltreatment and mental health problems in adulthood: Birth cohort study. Br. J. Psychiatry.

[20] Radford L., Corral S., Bradley C., Fisher H.L. (2013). The prevalence and impact of child maltreatment and other types of victimization in the UK: Findings from a population survey of caregivers, children and young people and young adults. Child Abus. Negl..

[21] Widom C.S., Widom C.S., DuMont K., Czaja S.J. (2007). A prospective investigation of major depressive disorder and comorbidity in abused and neglected children grown up. Arch. Gen. Psychiatry.

[22] Bowlus A., McKenna K., Day T., Wright D. (2003). The economic costs and consequences of child abuse in Canada: Report to the Law Commission of Canada. https://cwrp.ca/publications/economic-costs-and-consequences-child-abuse-canada.

[23] Conti G., Morris S., Melnychuk M., Pizzo E. (2017). The Economic Costs of Child Maltreatment in the UK.

[24] Fang X., Brown D.S., Florence C.S., Mercy J.A. (2012). The economic burden of child maltreatment in the United States and implications for prevention. Child Abus. Negl..

[25] Fang X., Fry D.A., Brown D.S., Mercy J.A., Dunne M.P., Butchart A.R., Corso P.S., Maynzyuk K., Dzhygyr Y., Chen Y. (2015). The burden of child maltreatment in the East Asia and Pacific region. Child Abus. Negl..

[26] Trocmé N., Esposito T., Fallon B., Chabot M., Delaye A., Lonne B., Scott D., Higgins D., Herrenkohl T.I. (2019). Building research capacity in child welfare in Canada: Advantages and challenges in working with administrative data. Re-Visioning Public Health Approaches for Protecting Children.

[27] U.S. Department of Health & Human Services, Administration for Children and Families, Administration for Children and Families, Administration on Children, Youth and Families, & Children’s Bureau (2022). Child Maltreatment 2020. https://www.acf.hhs.gov/cb/data-research/child-maltreatment.

[28] Provincial Directors (2020). Bilan des directeurs de la protection de la jeunesse/directeurs provinciaux 2020: Plus forts ensemble! Gouvernement du Québec. https://www.cisss-bsl.gouv.qc.ca/sites/default/files/fichier/bilan_dpj_2020_version_web.pdf.

[29] Provincial Directors (2019). Bilan des directeurs de la protection de la jeunesse/directeurs provinciaux 2019: Bilan des directeurs de la protection de la jeunesse/directeurs provinciaux 2019: 40 ans d’expertise pour bâtir l’avenir. Gouvernement du Québec. https://www.cisss-bsl.gouv.qc.ca/sites/default/files/fichier/1bilan2019_w_ste_web.pdf.

[30] Provincial Directors (2018). Bilan des directeurs de la protection de la jeunesse/directeurs provinciaux 2018: La cause des enfants tatouée sur le cœur. Gouvernement du Québec. https://www.cisss-bsl.gouv.qc.ca/sites/default/files/fichier/bilandpj_2018_vweb.pdf.

[31] Provincial Directors (2017). Bilan des directeurs de la protection de la jeunesse/directeurs provinciaux 2017: L’adolescence, une traversée en eaux vives. Gouvernement du Québec. https://www.santeestrie.qc.ca/uploads/media/Bilan_DPJ-DP_provincial_-_donnees_2016-2017_01.pdf.

[32] Provincial Directors (2017). Bilan des directeurs de la protection de la jeunesse/directeurs provinciaux 2016: Les mauvaises traitements psychologiques, un mal silencieux. Gouvernement du Québec. https://www.cisss-bsl.gouv.qc.ca/sites/default/files/fichier/bilan_2016_vfinale_taille_reduite_0.pdf.

[33] Public Health Agency of Canada (2020). Canadian Incidence Study of Reported Child Abuse and Neglect–2008: Major Findings. Ottawa, ON. https://cwrp.ca/sites/default/files/publications/CIS-2008-rprt-eng.pdf.

[34] Hélie S., Trocmé S., Collin-Vézina D., Esposito T., Morin S., Saint-Girons M. (2022). First Nations/Quebec Incidence Study of Child Maltreatment and Serious Behaviour Problems Investigated by Child Protection Services in 2019.

[35] Fallon B., Filippelli J., Lefebvre R., Joh-Carnella N., Trocmé N., Black T., MacLaurin B., Hélie S., Morin Y., Fluke J. (2020). Ontario Incidence Study of Reported Child Abuse and Neglect-2018 (OIS-2018).

[36] Mathews B., Pacella R., Dunne M.P., Simunovic M., Marston C. (2020). Improving measurement of child abuse and neglect: A systematic review and analysis of national prevalence studies. PLoS ONE.

[37] Kim H., Drake B. (2019). Cumulative prevalence of onset and recurrence of child maltreatment reports. J. Am. Acad. Child Adolesc. Psychiatry.

[38] Kim H., Wildeman C.J., Jonson-Reid M., Drake B. (2017). Lifetime prevalence of investigating child maltreatment among US children. Am. J. Public Health.

[39] Spano R. (2018). We are family: Specifying the unique contribution of abuse and neglect of siblings on the prevalence, severity, and chronicity of maltreatment in the household. J. Interpers. Violence.

[40] Prevoo M.J.L., Stoltenborgh M., Alink L.R.A., Bakermans-Kranenberg M.J., van Ijzendoorn M.H. (2016). Methodological moderators in prevalence studies on child maltreatment: Review of a series of meta-analyses. Child Abus. Rev..

[41] Seay K. (2015). How many families in child welfare services are affected by parental substance use disorders? A common question that remains unanswered. Child Abus. Rev..

[42] Finkelhor D. (1994). Current information on the scope and nature of child sexual abuse. Future Child..

[43] Lev-Wiesel R., Eisikovits Z., First M., Gottfried R., Mehlhausen D. (2018). Prevalence of child maltreatment in Israel: A national epidemiological study. J. Child Adolesc. Trauma.

[44] Wildeman C., Emanuel N. (2014). Cumulative risks of foster care placement by age 18 for U.S. children, 2000–2011. PLoS ONE.

[45] Stoltenborgh M., Bakermans-Kranenburg M.J., Alink L.R.A., van IJzendoorn M.H. (2015). The prevalence of child maltreatment across the globe: Review of a series of meta-analyses. Child Abus. Rev..

[46] Barth J., Bermetz L., Heim E., Trelle S., Tonia T. (2013). The current prevalence of child sexual abuse worldwide: A systematic review and meta-analysis. Int. J. Public Health.

[47] Finkelhor D., Shattuck A., Turner H.A., Hamby S.L. (2014). The lifetime prevalence of child sexual abuse and sexual assault assessed in late adolescence. J. Adolesc. Health.

[48] Government of Canada (2012). Child Maltreatment in Canada.

[49] Gaspard H. (2018). Enabling First Nations Children to Thrive.

[50] Turcotte D., Hélie S. (2012). Child protection policy reform in Quebec: Its effect on placement and stability in substitute care. Child Welf..

[51] Gouvernement du Québec (2021). Instaurer une Société Bienveillante Pour Nos Enfants et Nos Jeunes: Rapport de la Commission Spéciale Sur les Droits des Enfants et la Protection de la Jeunesse.

[52] Fallon B., Trocmé N., Black T., Ekins A., O’Connor C., Betito P.-A. (2017). Recurrence Rates by Urgent Protection and Chronic Need.

[53] Edwards F., Wakefield S., Healy K., Wildeman C. (2021). Contact with Child Protective Services is pervasive but unequally distributed by race and ethnicity in large US counties. Proc. Natl. Acad. Sci. USA.

[54] Fallon B., Lefebvre R., Trocmé N., Richard K., Hélie S., Montgomery H.M., Bennett M., Joh-Carnella N., Saint-Girons M., Filippelli J. (2021). Denouncing the Continued Overrepresentation of First Nations Children in Canadian Child Welfare: Findings from the First Nations/Canadian Incidence Study of Reported Child Abuse and Neglect-2019.

[55] Afifi T.O. (2011). Child maltreatment in Canada: An understudied public health problem. Can. J. Public Health.

[56] Sinha V., Ellenbogen S., Trocmé N. (2013). Substantiating neglect of first nations and non-aboriginal children. Child. Youth Serv. Rev..

[57] Putnam-Hornstein E., Ahn E., Prindle J., Webster D. (2021). Contact with the child protection system is pervasive, but are recent estimates correct?. Proc. Natl. Acad. Sci. USA.

[58] Grieger L.D., Danziger S.H. (2011). Who receives food stamps during adulthood? Analyzing repeatable events with incomplete event histories. Demography.

[59] Steensma C., Choi B.C.K., Loukine L., Schanzer D. (2018). Period Life Tables for Calculating Life Expectancy: Options to Assess and Minimize the Potential for Bias. Am. J. Public Health.

[60] Segal L., Nguyen H., Mansor M.M., Gnanamanickam E., Doidge J.C., Preen D.B., Brown D.S., Pearson O., Armfield J.M. (2019). Lifetime risk of child protection system involvement in South Australia for Aboriginal and non-Aboriginal children, 1986–2017 using linked administrative data. Child Abus. Negl..

[61] (2007). Youth Protection Act, R.S.Q. p-34.1. http://legisquebec.gouv.qc.ca/en/pdf/cs/P-34.1.pdf.

[62] Logan-Greene P., Semanchin Jones A. (2017). Predicting chronic neglect: Understanding risk and protective factors for CPS-involved families. Child Fam. Soc. Work..

[63] Maguire-Jack K., Font S.A. (2017). Intersections of individual and neighborhood disadvantage: Implications for child maltreatment. Child. Youth Serv. Rev..

[64] Rothwell D.W., Wegner-Lohin J., Fast E., de Boer K., Trocmé N., Fallon B., Esposito T. (2018). Explaining the economic disparity gap in the rate of substantiated child maltreatment in Canada (Part I). J. Law Soc. Policy.

[65] Ministère de la Santé et des Services Sociaux (2020). Your Child’s Situation Has Been Reported to the DYP: What Do You Need to Know?.

[66] Hélie S., Turcotte D., Trocmé N., Tourigny M. (2012). Étude d’Incidence Québécoise sur les Situations évaluées en Protection de la Jeunesse en 2008: Faits Saillants.

[67] Boatswain-Kyte A., Esposito T., Trocmé N. (2020). A longitudinal jurisdictional study of Black children reported to child protection services in Quebec, Canada. Child. Youth Serv. Rev..

[68] Dettlaff A.J., Weber K., Pendleton M., Boyd R., Bettencourt B., Burton L. (2020). It is not a broken system, it is a system that needs to be broken: The upend movement to abolish the child welfare system. J. Public Child Welf..

[69] Connell-Carrick K., Scannapieco M. (2006). Ecological correlates of neglect in infants and toddlers. J. Interpers. Violence.

[70] Hamilton L., Martin-West S. (2019). Universal basic income, poverty, and social justice: A moral and economic imperative for social workers. Soc. Work..

[71] Hyslop I., Keddell E. (2018). Outing the elephants: Exploring a new paradigm for child protection social work. Soc. Sci..

[72] Wiederspan J., Rhodes E., Shaefer H.L. (2015). Expanding the discourse on antipoverty policy: Reconsidering a negative income tax. J. Poverty.

[73] Best J. (1990). Threatened Children: Rhetoric and Concern about Child-Victims.

